# Wear Resistance Enhancement of Rotary Tillage Blades Through Structural Optimization and Surface Strengthening

**DOI:** 10.3390/ma18215006

**Published:** 2025-11-02

**Authors:** Zechang Zou, Jiacheng Li, Xingwang Wang, Cuiyong Tang, Xueyong Chen

**Affiliations:** 1 College of Mechanical and Electrical Engineering, Fujian Agriculture and Forestry University, Fuzhou 350002, China; zouzechang@fjcpc.edu.cn (Z.Z.); 17708427440@163.com (J.L.); 22412098002@fafu.edu.cn (X.W.); hnrtcy@163.com (C.T.); 2 School of Mechanical and Intelligent Manufacturing, Fujian Chuanzheng Communications College, Fuzhou 350007, China

**Keywords:** rotary tillage blade, energy efficiency, structural optimization, SPH simulation, laser cladding, wear resistance

## Abstract

Rotary tillage blades, as critical components of soil tillage machinery, encounter significant challenges in mountainous agricultural operations, where excessive wear and high energy consumption are persistent issues. To address these problems, this study proposes an integrated strategy combining structural optimization with surface reinforcement. A blade–soil interaction model based on Smoothed Particle Hydrodynamics (SPH) was developed to optimize blade geometry, reducing power consumption to 0.106 kW with a simulation error of only 2.83%. In parallel, Fe60–WC composite coatings containing 30%, 35%, and 40% WC were fabricated on 65Mn substrates using laser cladding. Microstructural analysis revealed significant grain refinement with increasing WC content, while tribological tests showed that the 35% WC coating blades exhibited superior wear resistance, with a mass loss of 1.9 mg, and a relatively low friction coefficient of 0.362. Field trials further confirmed that the blades resulted in a 45.75% reduction in average wear, after structural enhancement and the application of the optimized coating, with a measured loss of 2.259 g compared to the uncoated blades. These findings demonstrate the synergistic benefits of structural optimization and advanced surface engineering, providing an effective pathway to improve the durability and efficiency of rotary tillage blades in demanding field conditions.

## 1. Introduction

With the growing demand for higher agricultural productivity and more efficient resource utilization, mountainous farming has attracted increasing attention, which poses significant challenges due to its steep terrain and complex soil conditions. However, farming operations in such environments are constrained by high energy consumption and severe tool wear, particularly under the abrasive conditions created by dry sandy soils. These factors have become major barriers to improving agricultural efficiency [1,2]. Rotary tillers, widely used in mechanized farming, operate under highly dynamic and complex soil–tool interactions, posing a significant challenge to the direct measurement of power consumption under actual field conditions [3]. Consequently, numerical simulations combined with field experiments have been extensively adopted for power evaluation [4]. For example, Hu et al. developed a Smoothed Particle Hydrodynamics (SPH) model based on elasto-plastic constitutive laws to simulate soil–tool interactions in cohesive and non-cohesive soils, accurately capturing soil shearing and fracture behavior and providing valuable insights into tillage processes [5].

To address the issue of excessive power consumption, many studies have focused on optimizing blade geometry [6] and operational parameters [7], with structural optimization being a commonly adopted strategy for reducing energy losses [8]. Wang et al. used response surface methodology (RSM) to analyze the mixing behavior of soil and heavy metal passivators under different kinematic and structural parameters, successfully identifying the optimal combination for a triple-helix auger [9]. Mao et al., with the goal of studying the wear and resistance characteristics of rotating blades, constructed a model of the blade–soil interaction through the development of a coupled Discrete Element Method and Multi-Body Dynamics (DEM-MBD) simulation [10]. Li et al. employed SPH simulations and Taguchi method-based soil bin tests to optimize rotary tiller blades with varying geometries, validating the model’s predictive accuracy and achieving reduced energy consumption [11]. Similarly, Zhang et al. conducted SPH-based simulations to investigate blade–soil interactions and further optimized critical design parameters using orthogonal experiments and RSM. The optimal parameters identified included a bend angle of 30°, a side-cutting edge curvature direction angle of 51°, and a curvature angle of 120° [12].

Although simulation-based optimization has significantly improved structural strength and operational efficiency [13], tool wear remains a critical limitation in field applications [14]. To mitigate this, laser cladding has emerged as a promising surface enhancement technique for improving wear resistance and extending tool life [15]. Laser-cladded WC/Fe coatings have the advantages of low cost and high abrasion wear resistance [16]. In a related study, a “brick–mortar” biomimetic structure on 65Mn steel via laser cladding was applied to enhance the performance of the rotary blade, which achieving a 3.6-fold increase in hardness and a 33% reduction in wear compared to the substrate [17]. Chen L. further investigated the effect of nano-La2O3 content on WC–12Co coatings and found that 1.2% La2O3 significantly enhanced coating hardness to 1436 HV0.5 via grain refinement and second-phase strengthening—260% higher than the base material [18]. In summary, the structural optimization of rotary blades via simulation for reduced power consumption, combined with laser-cladded wear-resistant coatings, can significantly improve the performance and operational longevity of blades.

Despite these advances, limited research has focused on optimizing rotary blade structures using SPH-based soil–blade interaction models. In this study, an SPH interaction model was developed to analyze soil–blade mechanics, with RSM employed to optimize three structural parameters—bending radius, wrap angle, and bending angle—using power consumption as the objective function. The simulation results were validated by field trials. Additionally, Fe60-based coatings with different WC contents were fabricated by laser cladding on 65Mn substrates. The coating with the best performance was applied to optimized blades, and its wear resistance was further confirmed in field tests. This integrated approach highlights the significance of combining structural optimization with surface strengthening to reduce wear and improve the performance of rotary tillage blades in complex agricultural environments.

## 2. Materials and Methods

### 2.1. Structural Configuration and Working Principle of the Rotary Tillage Blade

**Figure 1:**
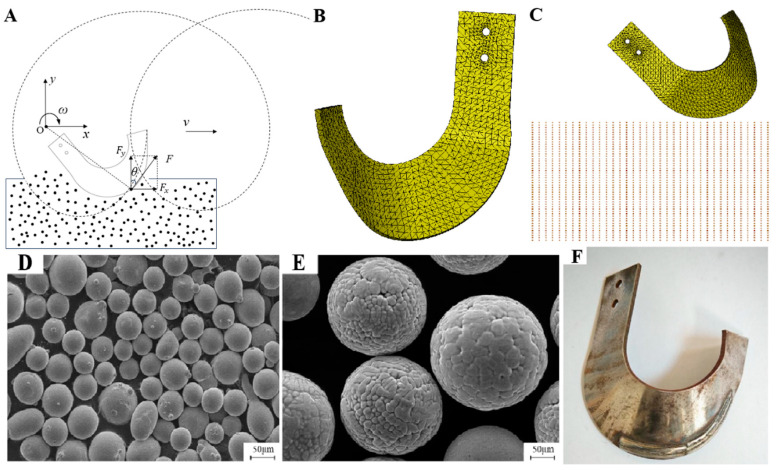
Trajectory, modeling, and preparation of rotary tiller blades: (**A**) trajectory and force of rotary tillage; (**B**) meshing of the rotary tiller blade model; (**C**) coupled tillage–soil model; (**D**) microscopic morphology of Fe60 alloy powder; (**E**) microscopic morphology of WC powder; (**F**): rotary tiller after laser cladding.

Figure 1A illustrates the motion trajectory and force analysis of a rotary tillage blade during soil cutting. The blade’s motion comprises two components: the forward motion of the rotary tiller and the circular motion of the blade around the blade shaft. The resulting trajectory follows a cycloidal path [19].

A Cartesian coordinate system is established with the blade shaft center denoted as point *O*, the horizontal axis as *x*, and the vertical axis as *y*. The position of point *P* on the blade at any time *t* can be expressed as

(1) $$x=\frac{-b\pm \sqrt{b^{2}-4ac}}{2a}\;x=R\cos(\omega t)+V_{m}t$$

(2) y=−Rsin(ωt)

where *R*: radius of the rotary blade (mm); *ω*: angular velocity of the blade (rad/s); *V_m_*: forward velocity of the tiller (m/s); and *t*: soil-cutting time (s).

During the soil-cutting process, the forces *F_x_* and *F_y_* act in the horizontal and vertical directions, respectively. These can be resolved as

(3) $$F_{x}=F\sin\theta$$

(4) $$F_{y}=F\cos\theta$$

where *F*: resultant force acting on point P of the blade (N); *F_x_*: horizontal component of the force (N); *F_y_*: vertical component of the force (N); and *θ*: angle between the resultant force and the vertical axis, defined as *θ* = arctan(*F_x_*/*F_y_*).

As these forces fluctuate periodically during the blade’s motion, and the torque *T* acting on the blade over time can be described by

(5) $$T=\int_{0}^{t0}\sqrt{F_{x}^{2}+F_{y}^{2}}L_{0}\sin\theta dt$$

where *T*: torque of the rotary blade (N·m); *t*0: total cutting duration (s); and *L*_0_: average contact length between the blade and soil along the cutting direction (m).

The power consumption *P* during cutting can then be expressed as

(6) $$P=T\omega +F_{x}V_{m}$$

where *P*: cutting power consumption (kW)

The total operational power consumption of the rotary tiller can be obtained using LS-PrePost 2021 simulation software [20]. The majority of this consumption arises from soil cutting resistance, which is influenced by variables such as rotational speed, soil adhesion resistance, blade curvature, and blade bending angle. Therefore, a comprehensive understanding of the underlying mechanics governing power consumption is essential for designing and optimizing the structural and operational parameters of rotary tillage blades [21].

### 2.2. Construction of the Tool–Soil Cutting Model and Experimental Design

#### 2.2.1. Construction of the Rotary Blade Model

In this study, a rotary tillage blade specifically designed for mountainous farming conditions was investigated. The blade was fabricated from 65Mn steel. To improve computational efficiency, a single blade group was selected for numerical simulation.

The model was then imported into Hyper Mesh 2021 for mesh generation. A mesh size of 2 mm was specified, while all other parameters were retained at their default system values. The resulting mesh consisted of 11,810 elements, as shown in Figure 1B. After meshing, material properties and operational parameters (e.g., tillage speed) were assigned to the blade model. The specific values of all simulation parameters are listed in Table 1.

#### 2.2.2. Construction of the Soil Model

The soil-cutting simulation was conducted using the Smoothed Particle Hydrodynamics (SPH) method. A soil model with dimensions of 160 mm × 240 mm × 100 mm was constructed. The particle spacing in the SPH soil model was set to one-fiftieth of the model’s smallest edge length to ensure sufficient resolution.

For the specific soil type selected in this study, measurements were performed on the test field (Fuzhou, China) using TPJSD-750-V soil compaction tester (Heilongjiang DEWO Technology Development Co., Ltd., Harbin, China). The test results indicated that soil properties such as bulk density, aggregate structure, moisture content, and porosity were closely aligned with those used in prior SPH-based simulations [22]. Therefore, the material properties of the soil in this study were defined accordingly, as summarized in Table 2. The fully coupled model of the rotary blade and soil domain is shown in Figure 1C.

#### 2.2.3. Response Surface Methodology (RSM)

In the simulation of soil cutting by rotary tillage blades, power consumption was selected as the response variable for optimization. A Box–Behnken Design (BBD) was employed within the framework of RSM for experimental design and data analysis [23]. Based on preliminary orthogonal experiments, three influential factors—bending radius, wrap angle, and bending angle—were selected for the BBD. The factor levels and experimental scheme are shown in Table 3.

### 2.3. Preparation of Laser-Cladded Coatings

#### 2.3.1. Coating Fabrication

In this experiment, Fe60 alloy powder (Zhongnuo New Materials (Beijing) Technology Co., Ltd., Beijing, China) with a particle size of 45–106 μm was used as the base cladding material. Hard WC particles (Zhongnuo New Materials (Beijing) Technology Co., Ltd., Beijing, China) were incorporated at mass fractions of 30%, 35%, and 40%, respectively. The powders were blended by mechanical ball milling under vacuum conditions. The milling process was conducted at a rotation speed of 150 r/min for 3 h. The chemical composition of the Fe60 powder is listed in Table 4 [24], and the microstructures of the Fe60 and WC powders are shown in Figure 1D and Figure 1E.

The coatings of the blade were detected by X-ray diffraction (XRD) with a Rigaku Ultima IV X-ray diffractometer (Rigaku Corporation, Tokyo, Japan). It uses a Kα-ray copper target, and its wavelength is λ = 1.54056 Å. Substrate samples were prepared from 65Mn steel plates with dimensions of 60 mm × 3 mm × 5 mm. Surface oxides were removed using an angle grinder. The surface roughness of the polished substrate was modified to Ra6.3–12.5 μm, followed by ultrasonic cleaning in absolute ethanol. The samples were then dried in a DZF-6090, vacuum oven (Zhengzhou Keda Machinery and Instrument Equipment Co., Ltd., Zhengzhou, China).

Laser cladding was carried out using a HL-WM-400-G2 system (DaZu Laser Technology Industry Group Co., Ltd., Shenzhen, China) to fabricate Fe60 coatings containing 30%, 35%, and 40% WC on the 65Mn substrates. The process parameters were as follows: laser power of 900 W, coaxial powder feeding rate of 7 g/min, scanning speed of 600 mm/min, overlap ratio of 25%, and three cladding tracks per sample.

#### 2.3.2. Microstructural Characterization and Wear Testing

The laser-cladded samples were sectioned via wire electrical discharge machining to obtain specimens measuring 8 mm × 8 mm × 5 mm, perpendicular to the cladding layer. Specimens were sequentially ground using 320#, 600#, 1200#, 1500#, and 2000# silicon carbide abrasive papers, followed by polishing with a 2000-mesh diamond paste on a metallographic polisher. The cross-sections were etched with aqua regia, cleaned, re-etched, and dried.

The phase composition of the cladding layers was identified using a Rigaku Ultima IV X-ray diffractometer (XRD). Microstructural observation was performed using a SU8010 high-resolution scanning electron microscope (SEM) (Hitachi High-Tech Corporation, Tokyo, Japan), and elemental analysis was carried out using its integrated energy-dispersive spectroscopy (EDS) system.

Vickers microhardness testing of the cladding cross-sections was conducted using an HMV-10S microhardness tester (Shimadzu Corporation, Kyoto, Japan). A load of 3000 g was applied for 10 s. Measurements were taken at 11 points along a path from the top surface of the cladding layer through the heat-affected zone (HAZ) to the substrate, with a spacing of 0.2 mm between adjacent points.

Reciprocating friction and wear tests were performed using an HSR-2R tribometer (Changzhou Ruipin Precision Instrument Co., Ltd., Changzhou, China) by reciprocating sliding tests against a SiC ceramic ball. Test parameters were as follows: a normal load of 10 N, a stroke length of 5 mm, a motor speed of 200 rpm, and a test duration of 40 min. The coefficient of friction and wear scar characteristics were recorded via the tribometer’s built-in system. Post-test, samples were ultrasonically cleaned in absolute ethanol for 15 min, vacuum-dried for 10 min, and weighed with a CP214 precision balance (Ohaus Instruments (Shanghai) Co., Ltd., Shanghai, China) to determine mass loss due to wear. Each sample underwent three rounds of repeatability tests. Due to the limitations of the experimental conditions, the test failed to measure the friction curve and wear marks of the surface coating of the rotary tiller in the real soil environment, but the test results have certain reference significance for evaluating the wear resistance of the rotary tiller in practical applications.

### 2.4. Field Validation Tests

#### 2.4.1. Power Consumption Measurement

To validate the accuracy of the rotary tiller simulation model, field experiments were conducted at the Qishan Campus of the Fujian Agriculture and Forestry University in Fuzhou, China. The soil properties at the site included a penetration resistance of 2–3 MPa, 15% gravel content by volume, 35% moisture content, and 37.8% porosity.

The test equipment was a handheld electric rotary tiller (Ningbo Senle Hardware Co., Ltd., Ningbo, China) powered by a 48 V 20 AH high-end lithium battery designed for landscaping tools. The motor operated at 220 V with a power output of 800 W and a maximum rotational speed of 8000 rpm.

Power consumption was determined by recording the battery discharge for each test run and measuring the duration of operation. Each tillage run was repeated three times, and the average value was taken as the final result. These data were compared with simulation results to verify the accuracy of the computational model.

#### 2.4.2. Wear Measurement Before and After Laser Cladding

Figure 1F shows the rotary tillage blade after laser cladding. The cutting edges of three optimized blades were coated with an Fe60 alloy containing 35% WC hard particles via laser cladding. Oxide layers on the blade surfaces were ground and polished prior to testing to ensure uniform surface conditions. The three cladded blades and three uncladded counterparts were randomly mounted on the same tiller shaft to ensure comparability.

After 3 h of operation under identical field conditions, the blades were removed and weighed to determine mass loss. This comparison served to evaluate the working performance and wear resistance of the cladded blades.

## 3. Results and Discussion

### 3.1. Analysis of Simulation Results

#### 3.1.1. Analysis of Blade Motion and Force Characteristics

**Figure 2:**
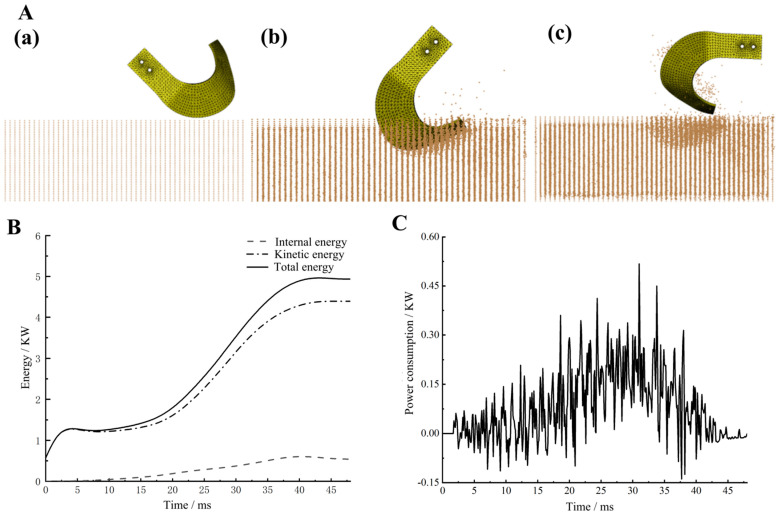
Cultivation knife–soil simulation motion process and energy analysis: (**A**) cultivation knife–soil simulation motion process: **A**(**a**) before entering the soil; **A**(**b**) entering the soil; **A**(**c**) after entering the soil; (**B**) time–energy relationship curve; (**C**) time–power consumption curve.

Figure 2A illustrates the corresponding blade–soil interaction states at representative points in time. By analyzing the simulation-based motion sequence in conjunction with the force profile acting on the blade, the interaction dynamics between the blade and soil particles at different time steps were assessed. At point A, the force acting on the blade is effectively zero, indicating that the blade has not yet made contact with the soil (see Figure 2(Aa). As the simulation progresses, the blade’s leading (tangential) edge and lateral (side-cutting) edge first come into contact with the soil. This is followed by contact between the blade’s lateral surface and the soil, generating significant compressive stress that displaces the soil particles along the blade’s surface. Consequently, the resistance force experienced by the blade begins to increase.

At point B, the force reaches its maximum. This peak occurs when the blade reaches its maximum penetration depth, resulting in the largest contact area between the blade and the soil. The maximum contact force corresponds to this maximum soil engagement, as illustrated in Figure 2(Ab). At point C, the blade just exits the soil, and the acting force sharply decreases to its minimum value. During this phase, the blade also expels fragmented soil particles outward due to the centrifugal effect upon soil detachment (see Figure 2(Ac). These simulation results are consistent with the findings reported by Zhang et al. [25], thereby reinforcing the validity of the model.

#### 3.1.2. Energy Analysis

Figure 2B illustrates the time–energy curve during the tillage process. Throughout soil cutting, the internal energy consumption remains relatively stable with minor fluctuations. As the blade comes into contact with the soil, the internal energy steadily increases, resulting in a corresponding rise in total energy consumption during the blade–soil interaction phase. Once the blade exits the soil, the energy consumption plateaus until the next contact event during the subsequent rotation cycle.

At approximately 4.5 ms, the internal energy reaches a local peak. This phenomenon is attributable to the energy conservation principle, where total energy is composed of internal and kinetic energy. An increase in kinetic energy at this stage leads to a temporary decrease in internal energy, maintaining the total energy balance.

Figure 2C shows the variation in power consumption over time. Due to the irregular distribution of soil particles and the stochastic nature of contact angles between the blade and fine soil elements, significant fluctuations in cutting power are observed during blade–soil contact. The power consumption reached its maximum at around 31 ms, coinciding with the blade’s deepest penetration into the soil. At this point, the cutting volume was at its highest, which explained the peak in power demand.

### 3.2. Analysis of RSM Results

To investigate the influence of rotary tillage blade structural parameters on soil-cutting power consumption, three key geometrical variables—bending angle (X_3_), bending radius (X_1_), and wrap angle (X_2_)—were selected as independent factors. An RSM was employed to determine the optimal combination of these parameters for minimizing power consumption.

The simulation-based experimental results for cutting power consumption are summarized in Table 5, and the corresponding analysis of variance (ANOVA) results are presented in Table 6.

The ANOVA indicates that the bending angle (X_3_) has the most statistically significant impact on power consumption, followed by the bending radius (X_1_) and wrap angle (X_2_). Each *p*-value in the ANOVA table represents the probability that the observed influence of a factor is due to random chance. Lower *p*-values indicate a higher level of statistical significance. Moreover, the interactions between any two factors (X_1_X_2_, X_1_X_3_, and X_2_X_3_) also exhibit a measurable effect on the power consumption. Based on the regression analysis of the response surface design, the regression equation for power consumption is obtained as follows:

Y = 0.111 + 0.0015X_1_ − 0.0021X_2_ + 0.0031X_3_ − 0.002X_1_X_2_ − 0.0017X_1_X_3_ + 0.0022X_2_X_3_ + 0.0019X_1_^2^ + 0.0005X_2_^2^ − 0.0025X_3_^2^ (7)

**Figure 3:**
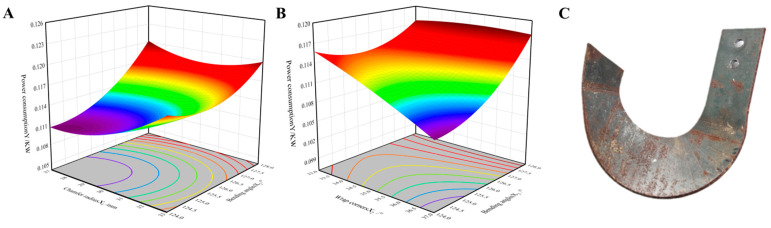
Analysis of response surface methodology (RSM) and the optimized solid model (**A**) the interaction result between bending angle radius and bending angle; (**B**) the results of the interaction between wrapping angle and bending angle; (**C**) optimized solid model of the rotary tiller blade.

Figure 3A illustrates the response surface of power consumption as a function of bending radius (X_1_) and bending angle (X_3_). The surface displays a steep gradient and densely packed, elliptical contour lines, indicating a strong interaction effect between X_1_ and X_3_ on power consumption. Specifically, within the bending angle range of 124–128°, power consumption increases with increasing bending radius. Observation of this response surface reveals that power consumption reaches a minimum when the bending radius is between 27 mm and 30 mm. Furthermore, as the bending angle decreases, power consumption is also reduced.

Figure 3B presents the response surface of power consumption with respect to the wrap angle (X_2_) and bending angle (X_3_). The relatively steep surface gradient again suggests a significant interaction between these two parameters. In the bending angle range of 124–128°, power consumption decreases as the wrap angle increases. Power consumption is minimized when the wrap angle lies between 36° and 37°. Similarly, a reduction in the bending angle contributes to a decrease in power consumption.

From the overall response surface analysis, it is evident that within the bending radius range of 27–32 mm, power consumption decreases with a reduction in X_1_. Likewise, in the wrap angle range of 35–37°, power consumption tends to decrease as X_2_ increases. To ensure the optimized blade structure is practical for manufacturing—minimizing unnecessary process adjustments and production costs—the final optimal parameter combination predicted by the model is

- Bending radius: 29 mm;
- Wrap angle: 37°;
- Bending angle: 124°.

Under these conditions, the predicted cutting power consumption is 0.106 kW.

SPH simulations were conducted for the optimized rotary blade structure. The simulation results yielded a power consumption of 0.109 kW. The relative error between the predicted value from the RSM model and the simulated result was 2.83%, indicating strong consistency among the prediction model, experimental design, and physical simulation. This confirms the validity of the optimization framework and the feasibility of the optimized design. The final manufactured rotary blade based on the optimal parameters is shown in Figure 3C.

### 3.3. Coating Performance Evaluation

#### 3.3.1. Microstructural Analysis

**Figure 4:**
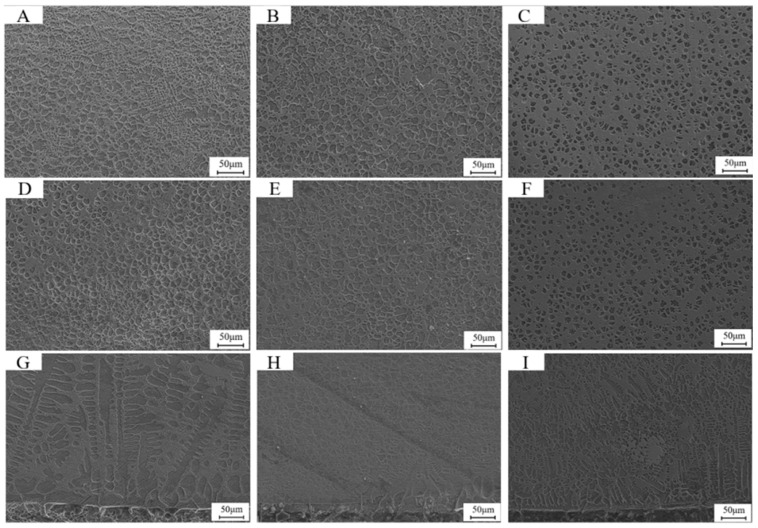
Microstructure of Fe60 alloy composite coatings with three different WC contents (**A**) 30% WC+Fe60 top cladding layer; (**B**) 35% WC+Fe60 top cladding layer; (**C**) 40% WC+Fe60 top cladding layer; (**D**) 30% WC+Fe60 middle cladding layer; (**E**) 35% WC+Fe60 middle cladding layer; (**F**) 40% WC+Fe60 middle cladding layer; (**G**) 30% WC+Fe60 bottom cladding layer; (**H**) 35% WC+Fe60 bottom cladding layer; (**I**) 40% WC+Fe60 middle cladding layer.

Figure 4 presents the microstructural characteristics in the top, middle, and bottom regions of the coatings fabricated with three different WC+Fe60 composite powder compositions. Longitudinally, the microstructure transitions from planar grains at the bottom to columnar dendrites in the middle and equiaxed grains at the top. This gradient is attributed to the varying ratio between the temperature gradient (G) and the solidification rate (R), which significantly influences solidification morphology.

At the bottom of the cladding layer, the interface between the molten alloy and the substrate experiences a relatively high temperature gradient due to rapid heat conduction into the substrate, while the cooling rate remains low. As a result, the G/R ratio is large, and the nucleation rate at the solid–liquid interface is lower than the growth rate. This leads to slow advancement of the interface and the formation of planar grains. As solidification progresses toward the surface, the temperature gradient gradually decreases while the solidification rate increases, resulting in a transition to coarser columnar dendrites.

At the top of the cladding layer, which is in direct contact with ambient air, the cooling rate is significantly higher, and the temperature gradient is smaller. The reduced G/R ratio promotes a nucleation rate that exceeds the growth rate, thereby favoring the formation of fine equiaxed grains [26].

In the transverse direction, a clear refinement trend is observed with increasing WC content. Specifically, the dendritic structures at the bottom evolve from coarse to fine dendrites. This refinement occurs because the addition of WC particles disrupts the vertical growth of dendrites, thereby limiting their growth length. At the middle and top regions, the equiaxed grains change from polygonal to near-spherical shapes, and the average grain size decreases significantly.

This microstructural refinement is attributed to the high melting point of WC particles compared to the Fe60 matrix. During the laser cladding process, WC particles create regions of locally increased undercooling. As undercooling increases, the nucleation rate becomes dominant over the growth rate, thus inhibiting grain coarsening and promoting fine grain formation [27].

**Figure 5:**
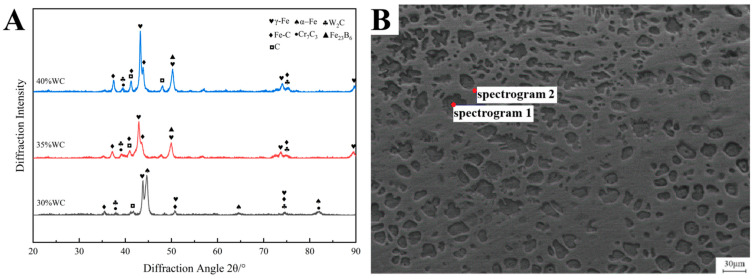
XRD diffraction patterns of the cladding layer with different proportions of WC hard-phase particles and EDS analysis positions of the top area of the cladding layer with 35% WC hard-phase particles (**A**) XRD diffraction patterns of the cladding layer with different proportions of WC hard-phase particles; (**B**) EDS analysis positions of the top area of the cladding layer with 35% WC hard-phase particles.

Figure 5A shows the X-ray diffraction (XRD) patterns of laser-cladded coatings with varying proportions of WC hard-phase particles. The diffraction data were analyzed using Jade 2019 software to identify the phase constituents.

The XRD results reveal that the coatings are primarily composed of phases including α-Fe, γ-Fe, W_2_C, Fe-C, Cr_7_C_3_, Fe_23_B_6_, and elemental carbon (C). When the WC content is 35%, the dominant phase is α-Fe. However, as the WC content increases, the dominant phase transitions from α-Fe to γ-Fe, with the relative intensity of γ-Fe increasing progressively. This trend is attributed to the thermal decomposition of WC under high-temperature laser irradiation, which releases carbon into the melt pool. The higher the WC content, the greater the concentration of carbon in the cladding layer.

Carbon, being a strong austenite stabilizer, enhances the thermodynamic stability of γ-Fe (austenite) and lowers the critical cooling rate required for the γ-Fe → α-Fe transformation. Consequently, the phase transformation is suppressed during solidification, resulting in a higher retained austenite fraction and a corresponding reduction in α-Fe peak intensity.

Moreover, the diffraction peaks of γ-Fe exhibit a noticeable leftward shift as the WC content increases. This shift is attributed to the formation of a Fe–C solid solution due to carbon enrichment in the matrix. The greater the WC content, the more abundant the carbon supply, promoting the formation of Fe–C phases. This is further evidenced by the increasing intensity of the diffraction peaks corresponding to the Fe–C phase.

Figure 5B and Table 7 show the selected EDS analysis points and corresponding elemental compositions on the top surface of the cladding layer containing 35 wt% WC particles.

From Table 7, it can be observed that Spectrum 1 exhibits a Ni content of 11.3 wt%. Nickel is a strong austenite-stabilizing element that expands the austenite phase field and suppresses the transformation from γ-Fe to α-Fe, thereby stabilizing γ-Fe at room temperature. In conventional austenitic stainless steels, the carbon content is typically limited to ≤0.08 wt%. However, under the non-equilibrium solidification conditions of laser cladding, local carbon enrichment can occur. In this case, the measured carbon content is elevated; a portion of the carbon is dissolved into the austenite matrix, further enhancing the stability of the γ-Fe phase. Moreover, the Cr content is comparable to that found in typical austenitic steels, supporting the identification of the austenitic phase. Combined with the XRD results in Figure 5A, the equiaxed grains at Spectrum 1 are confirmed to be predominantly γ-Fe.

In Spectrum 2, the Fe content is 93.20 wt% and the B content is 4.71 wt%. Atomic ratio calculations indicate that Fe and B are present in a stoichiometric ratio close to 23:6. Additionally, the contents of C, Cr, and W in this region are relatively low, suggesting that this area is dominated by iron–boride intermetallic phases. When cross-referenced with the XRD analysis from Figure 5A, the phase between the equiaxed grains in Spectrum 2 is identified as Fe_23_B_6_.

#### 3.3.2. Mechanical Properties of the Coating

**Figure 6:**
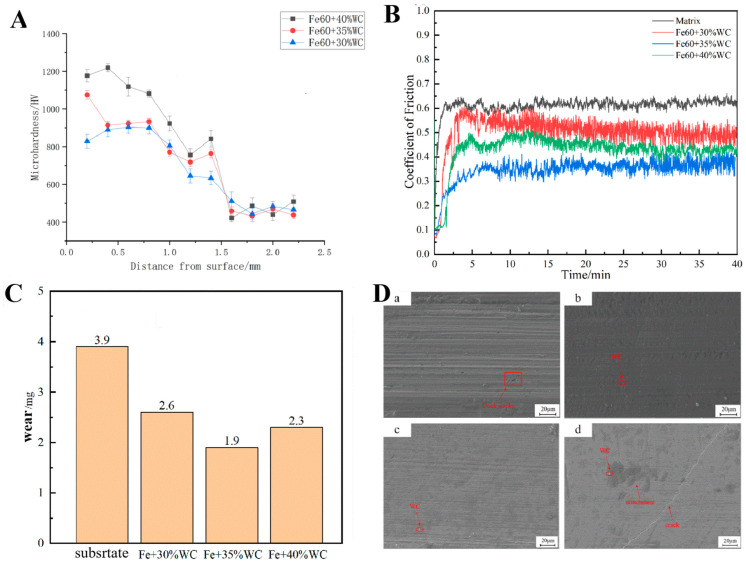
Microhardness distribution, friction coefficient, and wear and surface wear morphology of Fe60 composite cladding layer with different WC contents (**A**) microhardness distribution point line graph of Fe60 powder cladding layer with different WC contents; (**B**) friction coefficient curve of Fe60 powder cladding layer with different WC contents; (**C**) wear amount of Fe60 powder cladding layer with different WC contents; (**D**) surface wear morphology of Fe60 composite cladding layer with 65Mn matrix and different WC contents; **D**(**a**) 65Mn matrix; **D**(**b**): 30% WC+Fe60 laser cladding layer; **D**(**c**): 35% WC+Fe60 laser cladding layer; **D**(**d**) 40% WC+Fe60 laser cladding layer.

Figure 6A displays the microhardness profiles of laser-cladded Fe60 coatings containing 30%, 35%, and 40% WC particles. The results show that the average microhardness values of the coatings with 30%, 35%, and 40% WC are 880.685 HV, 961.175 HV, and 1148.684 HV, respectively. These values are approximately 1.9, 2.1, and 2.4 times higher than that of the 65Mn steel substrate. The highest hardness value observed was 1218.437 HV in the coating with 40% WC content.

Both the cladding zone and the heat-affected zone (HAZ) exhibit significantly higher hardness compared to the substrate. Furthermore, the average hardness of the cladding layer increases with increasing WC content [17]. This enhancement is primarily attributed to several strengthening mechanisms:

- Solid solution strengthening: During laser cladding, the WC hard phase partially dissolves, releasing carbon atoms that diffuse into the γ-Fe lattice. The increased solute concentration enhances solid solution strengthening.
- Second-phase strengthening: The released C and Cr elements form Fe–C solid solutions and Cr_7_C_3_ carbides, which contribute to second-phase strengthening. The presence of Cr_7_C_3_ also inhibits grain growth by reducing grain boundary mobility.
- Grain refinement strengthening: The rapid solidification under a steep thermal gradient in the melt pool promotes finer grain structures, leading to grain boundary strengthening.
- Dispersion strengthening: The Fe23B6 intermetallic compound, identified as a reinforcing phase [28], is uniformly distributed within the cladding matrix, thereby contributing to dispersion strengthening and improving overall hardness.

Additionally, in coatings with 35% and 40% WC, the HAZ near the substrate exhibits a noticeable increase in hardness. This is likely due to the rapid heat dissipation near the substrate, which produces a microstructure analogous to that formed during quenching. This refined grain structure further contributes to hardness enhancement. Notably, the peak hardness values are typically observed in the fine-grained regions adjacent to the substrate interface.

#### 3.3.3. Friction and Wear Performance Analysis

Figure 6B shows the time-dependent variation in the friction coefficient under a 10 N load for Fe60 composite coatings with different WC contents and for the 65Mn substrate. The average friction coefficients for the substrate, 30% WC composite coating, 35% WC composite coating, and 40% WC composite coating were 0.620, 0.448, 0.362, and 0.402, respectively. All three composite coatings exhibited significantly lower average friction coefficients than the substrate.

The evolution of the friction coefficient over time followed a similar trend across all samples and can be divided into two distinct phases: a run-in wear stage and a steady-state wear stage. During the initial 0–5 min, the system was in the run-in phase, where the friction coefficient increased rapidly. This behavior is primarily attributed to contact between the counterface and the relatively rough coating surface, where exposed WC particles hinder smooth sliding. In this stage, the actual contact area between the ceramic ball and coating is small, resulting in high localized shear stress and thus a sharp increase in the friction coefficient.

With the formation of plowing grooves, the contact between the friction pair and the coating changes from point contact to line contact and surface contact, and the degree of contact increases. The debris generated on the surface forms a transfer film with a certain bearing capacity, or the damaged WC particles are discharged from the plowing groove due to a plowing effect, which tends to stabilize the friction coefficient value. Additionally, during the steady-state phase, minor fluctuations in the friction coefficient were observed in all samples. These variations are attributed to heat generation at the contact interface, which can induce thermal stress and affect the mechanical properties of the cladding layer.

Figure 6C presents the wear mass loss for coatings with different WC contents. The wear losses for the Fe60 coatings with 30%, 35%, and 40% WC were 2.6 mg, 1.9 mg, and 2.3 mg, respectively—all lower than the substrate’s wear loss of 3.9 mg. The improved wear resistance is primarily due to the addition of WC hard particles, which promote grain refinement and dispersion strengthening within the cladding layer.

However, although increasing the WC content generally leads to reduced wear volume, an excessive amount of WC (i.e., 40%) resulted in increased wear. This phenomenon is attributed to the thermal decomposition of WC during the laser cladding process. The released carbon reacts with ambient oxygen at high temperatures, forming CO and CO_2_ gases. If these gases do not escape in time, they form pores within the coating. Under the applied load during friction, these pores can collapse, generating debris and accelerating material removal [29].

Furthermore, a high WC content leads to a larger mismatch in the thermal expansion coefficient between the coating and the 65Mn substrate. This mismatch induces thermal stresses, increasing the likelihood of crack formation in the coating. These cracks contribute to additional debris generation during sliding, further exacerbating the wear rate.

Figure 6D shows the surface wear morphologies of the 65Mn substrate and Fe60 composite coatings with different WC contents. As observed in Figure 6(Da), the 65Mn substrate exhibited deep grooves, furrow-like scratches, and adhered debris and pits after friction and wear testing. Due to the lack of laser cladding strengthening, the substrate undergoes certain plastic deformation under the action of friction pair and debris shear stress, forming a plowed groove morphology. During relative motion against the counterface, frictional heat raised the surface temperature significantly, resulting in a steep temperature gradient along the depth of the contact layer. The hardness of metallic surfaces tends to decrease with increasing temperature, thereby accelerating adhesive wear.

Additionally, thermal stress induced by this temperature gradient promotes plastic deformation of the contact layer. A greater gradient leads to more severe plastic deformation and, consequently, more intensive wear. Some wear debris becomes entrapped between the counterface and the sample, forming adhered layers under compressive stress. Therefore, the dominant wear mechanism for the 65Mn substrate is identified as adhesive wear [30].

In contrast, Figure 6D(b–d) demonstrate that with increasing WC content, the wear scars become shallower, and WC particles are observed on the surface. During the wear process, some WC particles detach from the coating and participate in sliding, producing fine scratches and causing minor accumulation of blackened debris. Thus, the wear mechanism of the coatings is characterized as mixed wear, involving both abrasive and adhesive wear.

Notably, Figure 6(Dd) reveals a visible crack, which is attributed to the significant mismatch in thermal expansion coefficients between the Fe60 matrix and the high WC content. This mismatch increases thermal stresses within the coating, making it more prone to crack initiation under frictional loading. These cracks, in turn, promote the formation of more debris and contribute to increased wear mass loss.

In summary, the mechanical performance evaluation of Fe60 coatings with varying WC content confirms that the 35% WC coating provides the optimal combination of properties. It exhibits significantly higher hardness than the 65Mn substrate and demonstrates the lowest friction coefficient and wear mass loss among the tested samples. Therefore, Fe60 powder containing 35% WC particles was selected for laser surface cladding reinforcement of the optimized rotary tiller blade.

### 3.4. Field Test Analysis

#### 3.4.1. Power Consumption Validation

Table 8 presents the field test results for power consumption. Under the optimized parameters determined from simulation, the rotary tiller was tested in three separate field trials. The results show that the average relative error between simulated and measured power consumption was 12.844%.

This deviation can be attributed to the complex conditions encountered during actual field operations, where soil is often mixed with small stones and plant roots. These obstacles increase the resistance encountered by the rotary blades, thereby elevating the power required for tillage. Nevertheless, this level of deviation is within acceptable limits for agricultural machinery evaluation, and the results validate the accuracy and feasibility of the simulation model.

#### 3.4.2. Wear Resistance Validation

The wear mass loss results for the two types of blades after 3 h of field operation are presented in Table 9. The average wear loss of the laser-cladded blades was 2.259 g, while that of the uncoated blades was 4.164 g. This corresponds to a 45.749% reduction in material loss for the coated blades, indicating a substantial improvement in wear resistance.

The enhanced wear performance can be attributed to the dense and refined microstructure formed by the rapid solidification characteristic of the laser cladding process. The presence of WC particles, Cr_7_C_3_ carbides, Fe–C solid solutions, and Fe_23_B_6_ borides in the cladding layer significantly improves the surface hardness and wear resistance of the rotary blade [31].

## 4. Conclusions

In this study, structural optimization and surface reinforcement were integrated to enhance blade efficiency. A coupled blade–soil interaction model was developed based on Smoothed Particle Hydrodynamics (SPH) to simulate tillage dynamics. The optimal parameter combination for reducing the power consumption of the rotary tiller blade structure was determined through response testing. Also, Fe60 alloy coatings incorporating varying proportions of WC (30%, 35%, 40%) were fabricated on 65Mn substrates via laser cladding, which was used to compare the average wear through field test. The following conclusions were obtained from this study:

1. A Smoothed Particle Hydrodynamics (SPH) model was established to simulate the rotary blade–soil interaction. The simulation effectively revealed the blade’s motion trajectory, force distribution, and power consumption during the tillage process. Based on the simulation results, the soil–blade interaction mechanism was analyzed in terms of blade movement and loading conditions.
2. Through RSM, the optimal structural parameters for minimizing tillage power consumption were determined as follows: bending radius = 29 mm, wrap angle = 37°, and bending angle = 124°. This resulted in a predicted power consumption of 0.106 kW. Simulation verification under these optimized parameters yielded a relative error of 2.830% between the predicted and actual simulated values. Field experiments further validated the simulation results, with an average relative error of 12.844% between the measured and simulated power consumption across three repeated trials, confirming the accuracy and feasibility of the SPH-based simulation model.
3. Fe60 composite coatings containing 30%, 35%, and 40% WC by mass were fabricated on 65Mn substrates via laser cladding. The primary phases identified in the coatings included α-Fe, γ-Fe, W_2_C, Fe–C, Cr_7_C_3_, Fe_23_B_6_, and elemental carbon. All coatings exhibited significant improvements in mechanical properties and wear resistance. The coating with 35% WC content demonstrated the best performance, with an average friction coefficient of 0.362 and a wear loss of 1.9 mg. The dominant wear mechanism was identified as a combination of abrasive and adhesive wear.
4. The Fe60 alloy powder containing 35% WC particles was selected for laser cladding reinforcement of the optimized rotary tillage blade edge. Field trials showed that the average wear mass of the cladded blades was 2.259 g, compared to 4.164 g for the uncoated blades—representing a 45.749% reduction in material loss. This confirms that the 35% WC Fe60 cladding significantly improves the wear resistance of rotary blades under actual field conditions.

## Figures and Tables

**Table 1:** Material properties and operational parameters of the rotary tillage blade parameters.

Parameter	Value
Material density (g·cm^3^)	7.85
Poisson’s ratio	0.30
Elastic modulus (GPa)	210
Forward velocity (m/s)	0.25
Rotational velocity (rad/s)	5

**Table 2:** Soil material properties.

Soil Material Properties	Value
Soil density (g·mm^−3^)	2.35 × 10^−6^
Porosity (%)	39.2
Soil bulk modulus (Pa)	4.65 × 10^6^
Moisture content (%)	34
Soil shear modulus (Pa)	1.86 × 10^7^
Viscoplastic parameters	1.1
Specific gravity of soil particles	2.79

**Table 3:** Factors and levels used in the response surface design.

Level	Bending Radius (mm)	Wrap Angle (°)	Bending Angle (°)
−1	27	33	124
0	30	35	126
1	33	37	128

**Table 4:** Chemical composition of Fe60 alloy powder. [24].

Element	C	Si	B	Cr	Ni	Fe
Content (wt%)	0.8–1.2	1.0–2.0	3.8–4.2	16–18	9.0–12	Balance

**Table 5:** Experimental design and results of the response surface methodology.

Run	Bending Radius (mm)	Wrap Angle (°)	Bending Angle (°)	Power Consumption (kV)
1	27	33	124	0.110
2	33	33	124	0.121
3	27	37	124	0.106
4	33	37	124	0.108
5	27	33	128	0.117
6	33	33	128	0.120
7	27	37	128	0.120
8	33	37	128	0.116
9	25	35	126	0.114
10	34	35	126	0.119
11	30	32	126	0.113
12	30	38	126	0.108
13	30	35	123	0.114
14	30	35	129	0.123
15	30	35	126	0.110
16	30	35	126	0.112
17	30	35	126	0.110
18	30	35	126	0.110
19	30	35	126	0.112
20	30	35	126	0.111

**Table 6:** Analysis of variance (ANOVA) for the response surface model.

Source	Sum of Squares	DF	Mean Square	F-Value	*p* Value	Significance
Model	0.0005	9	0.0001	44.214	<0.0001	***
X_1_	0.0000	1	0.0000	26.645	0.0004	**
X_2_	0.0001	1	0.0001	51.694	<0.0001	***
X_3_	0.0001	1	0.0001	113.437	<0.0001	***
X_1_X_2_	0.0000	1	0.0000	28.244	0.0003	**
X_1_X_3_	0.0000	1	0.0000	19.224	0.0014	**
X_2_X_3_	0.0000	1	0.0000	34.175	0.0002	**
X_1_^2^	0.0001	1	0.0001	44.191	<0.0001	***
X_2_^2^	0.0000	1	0.0000	2.597	0.1381	*
X_3_^2^	0.0001	1	0.0001	81.783	< 0.0001	***
Residual	0.0000	10	1.132 × 10^−6^	-	-	-
Lack of Fit	7.836 × 10^−6^	5	1.567 × 10^−6^	2.243	0.1980	*
Pure Error	3.493 × 10^−6^	5	6.987 × 10^−7^	-	-	-
Total	0.0005	19	-	-	-	-

Note: ***: extremely significant (*p* ≤ 0.0001); **: significant (0.0001 < *p* ≤ 0.05); *: not significant (*p* > 0.05).

**Table 7:** EDS analysis results of the top region of the coating with 35% WC hard-phase particles.

Region	C (wt%)	Fe (wt%)	Cr (wt%)	B (wt%)	W (wt%)	Ni (wt%)
Spectrum 1	1.27	63.84	14.8	2.84	0	11.3
Spectrum 2	0.49	93.20	0.23	4.71	1.37	0

**Table 8:** Results of field experiments.

Trial	Power Consumption (kW)	Relative Error (%)
1	0.125	14.679
2	0.121	11.009
3	0.122	11.927
Average	0.123	12.844

**Table 9:** Weight records of rotary blades before and after field testing.

	Uncoated Blade			Laser-Cladded Blade		
Sample lD	1	2	3	1	2	3
Weight before test (g)	125.783	125.586	125.780	128.760	129.820	129.195
Weight after test (g)	122.209	120.274	122.174	126.896	126.946	127.156
Wear loss (g)	3.574	5.312	3.606	1.864	2.874	2.039
Average wear loss (g)	4.164			2.259		

## Data Availability

The original contributions presented in this study are included in the article. Further inquiries can be directed to the corresponding author.
